# Divergent Resorbability and Effects on Osteoclast Formation of Commonly Used Bone Substitutes in a Human *In Vitro-*Assay

**DOI:** 10.1371/journal.pone.0046757

**Published:** 2012-10-10

**Authors:** Johannes Keller, Silja Brink, Björn Busse, Arndt F. Schilling, Thorsten Schinke, Michael Amling, Tobias Lange

**Affiliations:** 1 Department of Osteology and Biomechanics, University Medical Center Hamburg-Eppendorf, Hamburg, Germany; 2 Department of Anatomy and Experimental Morphology, University Medical Center Hamburg-Eppendorf, Hamburg, Germany; INSERM U1059/LBTO, Université Jean Monnet, France

## Abstract

Bioactive bone substitute materials are a valuable alternative to autologous bone transplantations in the repair of skeletal defects. However, clinical studies have reported varying success rates for many commonly used biomaterials. While osteoblasts have traditionally been regarded as key players mediating osseointegration, increasing evidence suggests that bone-resorbing osteoclasts are of crucial importance for the longevity of applied biomaterials. As no standardized data on the resorbability of biomaterials exists, we applied an *in vitro*-assay to compare ten commonly used bone substitutes. Human peripheral blood mononuclear cells (PBMCs) were differentiated into osteoclasts in the co-presence of dentin chips and biomaterials or dentin alone (control) for a period of 28 days. Osteoclast maturation was monitored on day 0 and 14 by light microscopy, and material-dependent changes in extracellular pH were assessed twice weekly. Mature osteoclasts were quantified using TRAP stainings on day 28 and their resorptive activity was determined on dentin (toluidin blue staining) and biomaterials (scanning electron microscopy, SEM). The analyzed biomaterials caused specific changes in the pH, which were correlated with osteoclast multinuclearity (r = 0.942; p = 0.034) and activity on biomaterials (r = 0.594; p = 0.041). Perossal led to a significant reduction of pH, nuclei per osteoclast and dentin resorption, whereas Tutogen bovine and Tutobone human strikingly increased all three parameters. Furthermore, natural biomaterials were resorbed more rapidly than synthetic biomaterials leading to differential relative resorption coefficients, which indicate whether bone substitutes lead to a balanced resorption or preferential resorption of either the biomaterial or the surrounding bone. Taken together, this study for the first time compares the effects of widely used biomaterials on osteoclast formation and resorbability in an unbiased approach that may now aid in improving the preclinical evaluation of bone substitute materials.

## Introduction

The repair of skeletal defects caused by trauma, infection or osteolytic bone metatases is one of the main clinical challenges of reconstructive surgery. For over 100 years the gold standard for repair has been the transplantation of autologous bone from the iliac crest, providing the advantages of both osteoconductive and osteoinductive action at the implanation site [1]. However, this procedure is hindered by donor site morbidity and limited availability [2].

As an alternative, readily available and inexpensive allografts and xenografts have been developed, however they bear the risk of impaired integration into the surrounding bone tissue and, more importantly, may induce immunologic responses in the recipient with unpredictable consequences [3], [4]. Therefore, synthetic bone substitutes composed of bioinert compounds such as metals or ceramics, have been used over several decades with the primary aim of mechanically augmenting skeletal defects [5]. In contrast to healthy bone tissue which constantly adjusts its structure to varying environmental needs [6], these materials cannot be remodeled, often resulting in material fatigue, loosening and implant failure. Hence, an important characteristic of synthetic bone substitutes is their bioactivity, which should ideally allow a defined resorption that is balanced with the speed of new bone formation, resulting in true osseointegration and *restitutio ad integrum* [7], [8]. In this context it is important to state that so called “resorbable” bone substitutes, including calcium phosphates, calcium sulphates, and calcium carbonates, have been somewhat unsuccessful as they were still detectable years after implantation due to impaired resorption. In contrast, some studies demonstrated accelerated resorption of several biomaterials which was not compensated by increased formation of new bone [9]. Thus, the choice of the biomaterial applied needs to be made according to patient comorbidities, such as low-turnover or high-turnover osteoporosis, in order to provide the best possible care with excellent outcomes. In this regard it is of utmost importance to test biomaterials preclinically, examining their resorbability by bone resorbing osteoclasts.

We have previously reported an *in vitro* assay using human osteoclasts that can be co-cultivated with dentin (ivory), as a model of physiological bone, and any biomaterial of interest. Using this assay we were able to examine the differentiation of human osteoclasts, as well as osteoclast activity in the presence of polymethylmetacrylate (PMMA) or calcium phosphate cement (Biobon) [9]. An analysis of the effects on osteoclast formation and resorbability from a range of biomaterials widely used clinically with a human cell-based assay has not been performed to date. Here, we have analyzed ten clinically used biomaterials for their influence on the differentiation and activity of human osteoclasts, and investigated whether the respective biomaterials exhibit an altered resorbabilty compared to dentin.

## Results

### Assay of Osteoclast Formation

**Figure 1:**
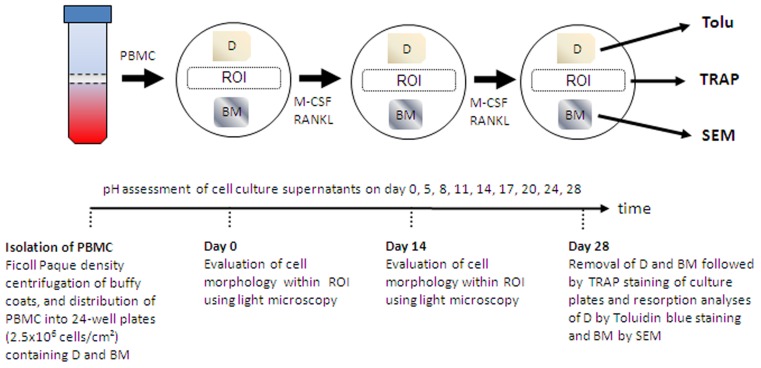
Generation of osteoclasts from peripheral blood mononuclear cells (PBMC) in the presence of dentin (D) and biomaterials (BM). Isolated PBMC were differentiated in the presence of Macrophage Colony-Stimulating Factor (M-CSF) and Receptor Activator of NF-κB Ligand (RANKL) for 28 days. 50% of the culture medium was changed every other day. Evaluation of cell morphology and quantification of mature osteoclasts was performed within the indicated region of interest (ROI). Culture wells containing exclusivley D were used as controls. Tartrate resistant acid phosphatase, TRAP; scanning electron microscopy, SEM; toluidine blue, Tolu.

**Figure 2:**
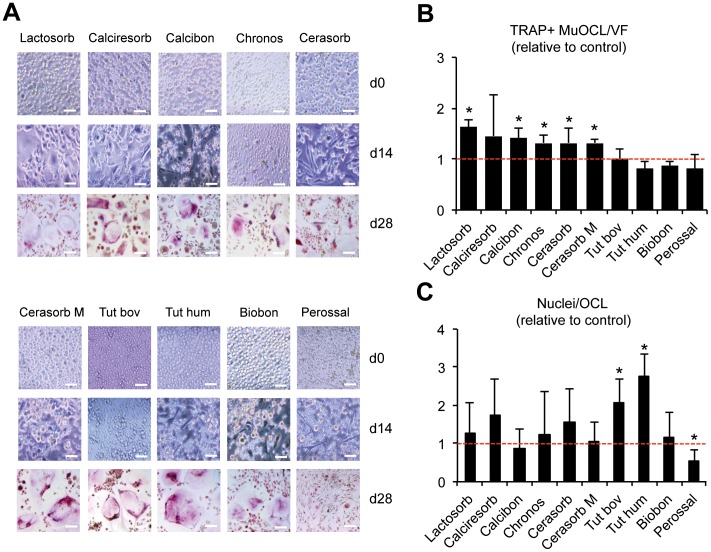
Osteoclast differentiation in the presence of various biomaterials. A) Light microscopy images of human osteoclast cell cultures at day 0, 14 (native) and 28 (TRAP stained ) in the presence of various clinically used biomaterials. Scale bars 25 µm. B) Quantification of TRAP positive multinucleated (>3 nuclei per cell) osteoclasts per viewing field (TRAP+ MuOCL/VF) in the same cultures. C) Quantitative determination of number of nuclei per counted osteoclast (Nuclei/OCL). Control is indicated as a dotted red line. Bars represent mean ± SD relative to control of four independent experiments with PBMCs from two different donors. Asterisks indicate statistically significant differences (p<0.05 vs. control).

The first aim of this study was to evaluate the differentiation of human osteoclasts in the co-presence of dentin as a model of physiologic bone and several bone substitute biomaterials widely used in clinical practice (Fig. 1). Starting with homogenous concentrations and distributions of mononuclear precursors in all groups on day 0 (light microscopy, Fig. 2A, upper panel) the presence of Calcibon, Cerasorb, Cerasorb M, Chronos and Lactosorb slightly increased the numbers of adherent cells with enlarged cellular protrusions in comparison to the control group with just dentin until day 14 (light microscopy, Fig. 2 A, middle panel). In contrast, the presence of Tutobone human, Perossal and Biobon did not alter osteoclast formation (Fig. 2 A). This discrepancy was much more pronounced on day 28, when TRAP positive, multinucleated osteoclasts were counted after staining (Fig. 2 A, lower panel). Calcibon, Calciresorb, Cerasorb, Cerasorb M, Chronos and Lactosorb led to a 1.3 to 1.6-fold increase in mature osteoclasts compared to controls (p<0.05), while the number of nuclei per osteoclast was not significantly altered (Fig. 2 B, C). Although osteoclastogenesis was not affected by the presence of Tutogen bovine and Tutobone human, both biomaterials led to a robust increase in the number of nuclei per osteoclast (p<0.05). In contrast, a significant reduction in osteoclast multinuclearity could be detected in the presence of Perossal.

### Assay of Resorbability

**Figure 3:**
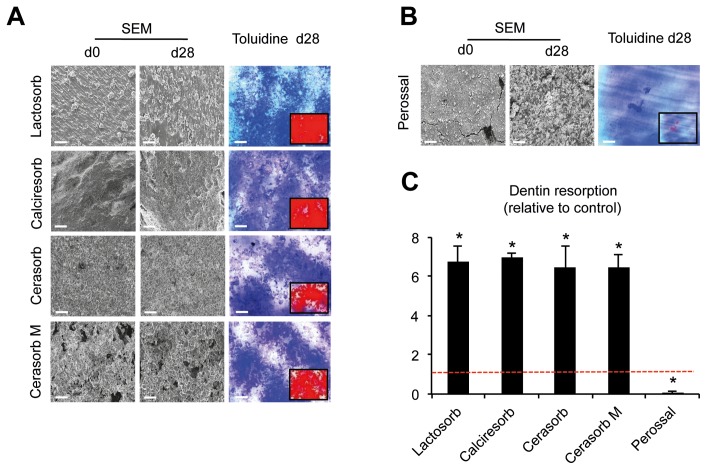
Dentin resorption in the presence of non-measurable biomaterials. A) and B) Representative scanning electron microscopy (SEM) images of selected biomaterials at day 0 and 28 (left panel) and toluidine blue stained dentin chips on day 28 (right panel) of osteoclast differentiation. Scale bars 50 µm. Note the irregular and coarse surface of biomaterials at both time points, making quantification of resorption impossible. C) Quantification of dentin resorption (mean resorption per osteoclast) cultured in the presence of biomaterials. Control is indicated as a dotted red line. Bars represent mean ± SD relative to control of four independent experiments with PBMCs from two different donors. Asterisks indicate statistically significant differences (p<0.05 vs. control).

**Figure 4:**
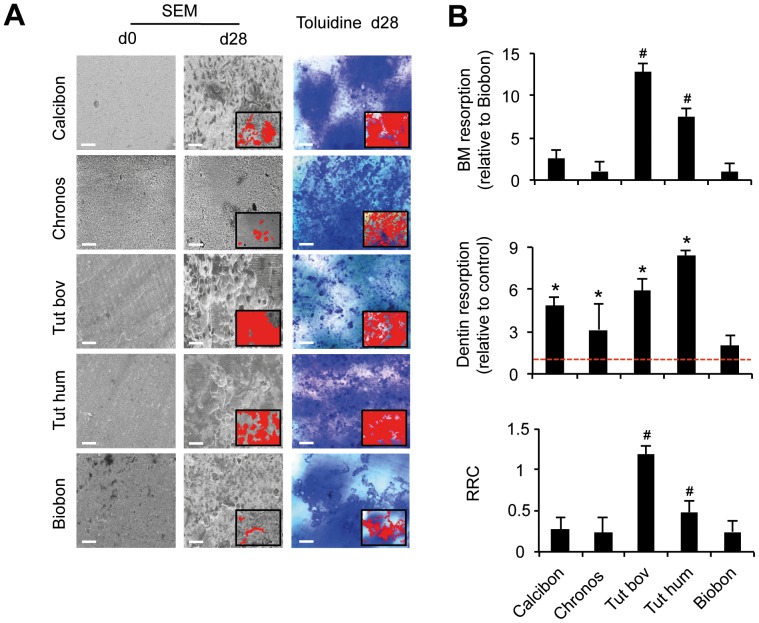
Resorption of biomaterial and dentin and determination of the RRC. A) Representative scanning electron microscopy (SEM) images of assessable biomaterials at day 0 and 28 (left panel) and toluidine blue stained dentin chips on day 28 (right panel) of osteoclast differentiation. Scale bars 50 µm. Note the smooth and regular surface of biomaterials at day 0, allowing precise determination of biomaterial resorption after 28 days of osteoclast culture. B) Quantification of biomaterial (BM, upper panel) and dentin (middle panel) resorption (mean resorption per osteoclast) and calculation of the relative resorption coefficient (RRC). Control is indicated as a dotted red line. Bars represent mean ± SD of four independent experiments with PBMCs from two different donors. Superscripts indicate statistically significant differences (*p<0.05 vs. control; ^#^p<0.05 vs. Biobon).

The second aim of this study was to determine potential effects of biomaterials on osteoclast activity. Therefore, the resorbed areas on dentin chips were quantified and normalized to the number of osteoclasts after 28 days of differentiation in the presence or absence (control) of the respective biomaterials. Interestingly, the vast majority of biomaterials (Calcibon, Calciresorb, Cerasorb, Cerasorb M, Lactosorb, Tutobone human and Tutogen bovine) caused a 3- to 8.5-fold increase in dentin resorption per osteoclast relative to control (p<0.05, Fig. 3 A +4 A). In contrast, the presence of Perossal almost completely impeded osteoclast activity on dentin (p<0.05, Fig. 3 C), whereas Biobon did not significantly alter the resorptive activity on dentin (Fig. 4 B, middle panel).

In the next step, resorption lacunae on biomaterials were quantified using scanning electron microscopy (SEM). Importantly, some of the materials revealed a rough, irregular or coarse surface at the beginning of the experiment (Lactosorb, Calciresorb, Cerasorb, Cerasorb M: Fig. 3 A; Perossal: Fig. 3 B), so that it was virtually impossible to differentiate, which changes of the material surface might have been due to osteoclast activity in these cases. Therefore, only materials with a smooth, uniform surface on day 0 were subjected to SEM-based quantifications of resorption on day 28 (Calcibon, Chronos, Tutogen bovine, Tutobone human, Biobon, Fig. 4 A). In accordance with its insignificant effect on dentin resorption, Biobon revealed only a marginal resorbability. In contrast, although all other materials had significantly increased the osteoclast activity on dentin, only Tutogen bovine and Tutobone human demonstrated an increased resorbability (12.5-fold and 7.5-fold, respectively, p<0.05, Fig. 4 B, upper panel), whereas the resorbability of Calcibon and Chronos was comparable to that of Biobon.

For those materials where quantification of resorption was possible, the relative resorption coefficients were calculated. For this subset of materials, the RRC was 1.19±0.11 (Tutogen bovine), 0.48±0.13 (Tutobone human), 0.28±0.15 (Calcibon), 0.25±0.14 (Biobon) and 0.23±0.18 (Chronos) (Fig. 4 B), respectively.

### Biomaterial-dependent Change in pH in Cell Culture Supernatants

**Figure 5:**
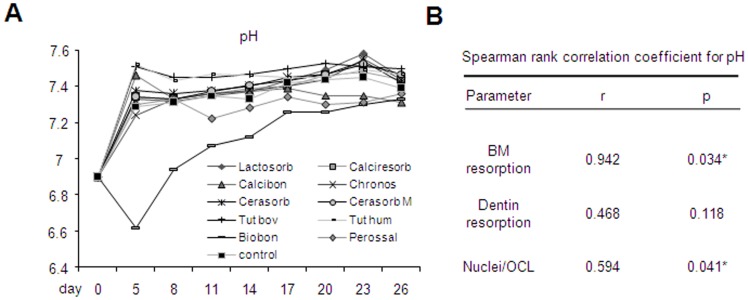
Biomaterial-dependent pH-value in cell culture supernatants. A) pH values during osteoclast differentiation in the presence of analyzed biomaterials. B) Spearman rank correlation coefficients (r) and respective p values of associations of biomaterial (BM) and dentin resorption as well as Nuclei/OCL with pH values are indicated. Asterisks indicate statistically significant differences (p<0.05).

Since the extracellular pH of culture medium has been reported to influence osteoclast formation and function [9], [10], we additionally assessed changes in pH during the culture period. Based on a pH of 6.9 at the beginning of incubation (day 0) the pH in each biomaterial group except Biobon increased to about 7.3 to 7.5 within the first 5 to 8 days of differentiation and did not change significantly until the end of the experiment (Fig. 5 A). Importantly, the strongest increase was found in the presence of Tutogen bovine and Tutobone human (pH = 7.5) and an interim decrease was observed in the Perossal group (with pH values lower than 7.3 between day 9 and 15). In contrast, Biobon caused an initial decrease in pH to a minimum of 6.6 after 5 days, followed by a slow increase to about 7.3 by day 18 of culture. Interestingly, there was a robust, statistically significant correlation between the pH value and the number of nuclei per osteoclast (r = 0.594, p = 0.041, Spearman rank test, Fig. 5 B). Likewise, the resorption on biomaterials was found to correlate significantly with the pH value (r = 0.942, p = 0.034, Spearman rank test, Fig. 5 B).

## Discussion

Given steadily improving surgical techniques, bone substitute materials are increasingly used to achieve *restitutio ad integrum* in patients with bone defects. However, despite the increasing use of synthetic biomaterials, contrary results regarding osseointegration have been reported and no applicable guidelines regarding the choice of appropriate bone substitutes exist [5]. Therefore, this study for the first time employed a standardized method to characterize the effects on osteoclast formation and resorbability of commonly used bone substitutes *in vitro*.

Most biomaterials analyzed in this study led to significant changes in osteoclast formation, as assessed by morphological analyses of maturing mononuclear precursors and counting of TRAP positive multinucleated osteoclasts and nuclei at the end of the culture period. While several biomaterials caused an increase in the formation of mature osteoclasts, this effect was not observable in the case of Calciresorb, Tutogen bovine, Tutobone human, Biobon and Perossal. Tutogen bovine and Tutobone human led to significantly increased nuclei per osteoclast, whereas less nuclei per cell were observed in the case of Perossal. As the extracellular pH of culture medium has been reported to be a significant determinant of osteoclastogenesis and osteoclast function [9], [10], we included pH measurements in our analysis. Per our previous findings, we chose a pH of 6.9 at the beginning of osteoclast differentiation. Importantly, the presence of Biobon resulted in a remarkable acidification (pH 6.6–7.1) of culture supernatants, whereas Perossal led to a marginal acidification (pH 7.2) and all other materials to a neutralization (pH 7.3–7.5) after 5 days of culture. Taken together, these findings demonstrate a significant correlation between pH and the number of nuclei per osteoclast. In accordance with the reduced number of nuclei per osteoclast, dentin resorption was significantly inhibited in the presence of Perossal. In contrast, Tutogen bovin and Tutobone human led to an increased number of nuclei per osteoclast and increased dentin resorption, which reflects the good osseointegration and smooth assimilation of bovine and human bone substitutes demonstrated in clinical studies [11].

In the next step, potential differences in the resorbability of the respective materials were investigated using SEM. Among all biomaterials analyzed we found Lactosorb, Calciresorb, Cerasorb, Cerasorb M and Perossal were not assessable by SEM due to their coarse and irregular surface already present at the beginning of the experiment. Hence, it was virtually impossible to differentiate which changes of the material surface might have been due to osteoclast activity in these cases. Therefore, alternative methods like confocal laser scanning microscopy or infinite focus microscopy, as well as quantitative determination of biomaterial-degradation products in culture supernatants may have to be applied in order to quantify osteoclast resorption [12]–[14]. However, among those biomaterials allowing quantitative determination of resorption, Tutogen bovine displayed the highest resorbabilty, followed by Tutobone human. This is in line with clinical studies confirming a sufficient replacement of the respective biomaterial by endogenous bone tissue [11], [15], [16]. As our morphologic analysis demonstrated no increase in mature osteoclasts, which could have explained the increased resorption of Tutogen bovine and Tutobone human, the number of nuclei appears to be a pivotal determinant of osteoclast resorption. These findings support several studies reporting a positive correlation between the number of nuclei per osteoclast and the resorptive capabilities of osteoclasts [17]. Clinically, this phenomenon is observable in the case of Paget’s disease of bone, where the formation of highly nucleated osteoclasts is accompanied by excessive bone resorption [18].

In contrast to Tutogen bovine and Tutobone human, Biobon showed only a very limited resorbability. Again, using spearman rank coefficient analysis, we revealed a significant and positive correlation between the material-dependent extracellular pH changes and biomaterial resorption. Although Biobon was postulated to be a good alternative to autologous bone transplants, non-degraded biomaterial was still detectable one year after implantation *in vivo* [19] and after 9–40 months in patients [20]. This limited resorption was also observable in our study and serves as a good control for the clinical relevance of the applied assay.

As it seems unlikely that the differences in dentin and biomaterial resorption were exclusively caused by differential effects of the materials on osteoclastogenesis, they may at least in part be explained by modulating effects on osteoclast activity. In this context, it is important to state that proinflammatory cytokines, such as TNF-alpha and Interleukin 6, have been reported to act as potent inductors of osteoclast activity [21], [22]. Similarly, we have previously demonstrated a considerable release of cytokines by the mononuclear cell fraction of peripheral blood in the presence of calcium phosphate based biomaterials [23], [24]. As the whole fraction of mononuclear cells was also used in the present study, it is tempting to speculate that a differential release of cytokines may have occured in the presence of the respective calcium phosphate based materials, contributing to the differential dentin and biomaterial resorption.

The quantification of osteoclastic resorption on biomaterials of interest often varies as no standardized method for differentiation of osteoclasts exists. Therefore, absolute resorption parameters differ between studies due to alternative protocols for osteoclast generation, cultivation and differentiation. To circumvent this problem and to make resorbability assays comparable, we have previously introduced the standardized parameter *relative resorbability coefficient* (RRC), which represents the quotient between resorption on biomaterial and resorption on dentin [9]. The RRC offers the advantage of eliminating differences in widely used protocols for the generation of functional osteoclasts, since resorption of the biomaterial is normalized to that of dentin. Furthermore, it provides additional information on whether biomaterials release substances or degradation products into the culture medium, influencing osteoclast formation and function. Due to the coarse and irregular surface of some biomaterials, only Tutobone human, Tutogen bovin, Calcibon, Biobon and Chronos could be compared for the RRC. While Tutobone human and Tutogen bovine displayed the highest absolute resorption, we also detected the largest RRC for these bone substitutes compared to Calcibon, Chronos and Biobon. Most interestingly however, the quantitative difference between biomaterial and dentin resorption in the case of Calcibon and Biobon was annihilated when calculating for the RRC. Thus, although Calcibon showed consistently higher percentages of biomaterial resorption and dentin resorption than Biobon, the RRC of both materials was almost equal. Likewise, while the presence of Calcibon and Chronos appears to stimulate osteoclast activity on dentin, both materials display limited resorbability (RRC<0.5), suggesting a preferential resorption of surrounding bone tissue in the case of Calcibon and Chronos.

Although the model applied in this study provides an important basis for the pre-clinical evaluations of bone substitutes regarding their effects on osteoclastogenesis and resorbability, we were not able to accurately determine total resorption on biomaterials and dentin chips. Due to the technical limitations of the applied methods it was only possible to determine the resorbed area, whereas the depth of resorption pits could not be assessed and requires measurement by infinite focus microscopy in future analyses [12]–[14]. Moreover, while the applied model is suitable for the analysis of osteoclast formation and resorbability, no information regarding osteoinductive characteristics of biomaterials can be yielded. However, this is of clinical importance since resorbed biomaterial should ideally be completely replaced by endogenous bone resulting in a true *restitutio ad integrum*. While several *in vitro* co-culture systems could be established imitating the bidirectional communication between osteoclasts and osteoblasts, there are no models for simultaneous analyses of osteoconductive and osteoinductive properties of bone substitutes [25], [26]. Therefore, novel culturing techniques are required to allow the determination of resorbability and osteoinductivity of selected biomaterials in a standardized assay.

In conclusion, this study compared the effects on osteoclast formation and resorbabilty of commonly used biomaterials using a human cell-based assay. Using this standardized and unbiased approach, we found a significant correlation between initial material-dependent changes in the pH of culture supernatants and osteoclast multinuclearity, as well as biomaterial resorption. Our assay is of potential clinical relevance, as the results are in line with previous reports on the resorbability of some of the materials *in vivo* and in patients. Moreover, our study defined relative resorption coefficients, which may now aid in improving preclinical assessment of bone substitute materials.

## Materials and Methods

### Isolation of Human Peripheral Blood Mononuclear Cells

Peripheral blood mononuclear cells (PBMC) containing the fraction of CD14+ precursors of human osteoclasts were isolated from buffy coats of healthy, consenting donors by density centrifugation using Ficoll Paque Plus (Amersham Biosciences, Uppsala, Sweden) as described before [9], [23], [24]. The buffy coats were generously provided by Dr. T. Kühnl (Department of Transfusion Medicine, University Medical Center Hamburg-Eppendorf). PBMC were cultured in 24-wells at a density of 2.5×10^6^ cells/cm^2^ in alpha-MEM (Sigma, Deisenhofen, Germany) containing 0.22% sodium bicarbonate (pH = 6.9 [9]), 10% FCS (Cambrex Biosciences, Verviers, Belgium), 1% penicillin/streptomycin (Gibco, Rockville, USA), 25 ng/mL M-CSF and 40 ng/mL RANKL (both from Peprotech, London, UK) at 5% CO_2_ and 95% H_2_O saturation. After 24 h of incubation, non-adherent cells were washed off to dispose the culture of contaminating lymphocytes. Human osteoclasts were differentiated in the presence of biomaterials and dentin for a period of 28 days by changing 50% of cell culture medium every other day (Fig. 1).

### Biomaterials and Dentin

Ten commonly used bone substitute materials were analyzed (Lactosorb, Calciresorb, Calcibon, Chronos, Cerasorb, Cerasorb M, Tutogen bovine, Tutobone human, Biobon, and Perossal) [15], [16], [21], [27]–[35]. The origin, chemical composition and clinical use of the biomaterials tested in this study are summarized in Table 1. Dentin (ivory) was kindly provided by German customs in accordance with the international laws for the protection of species. All materials were cut into small chips ranging from 5×3×1 mm (dentin, Tutogen bovin, Tutobone human, Cerapatite) to 5×5×2 mm (Calciresorb, Perossal, Lactosorb, Cerasorb, Cerasorb M) using a sterile scalpel or low speed saw. Biobon, Calcibon and Chronos were compounded from a liquid and a powdery component according to the manufacturer’s instructions and were cut into appropriate chips after hardening of the composites. Prior to incubation with PBMC, all materials were analyzed in a raster electron microscope as described below and were then transferred to the corresponding 24-wells.

**Table 1:** Basic characteristics of analyzed biomaterials.

Biomaterial	Composition	Manufacturer	Clinical studies
Lactosorb	L-Lactic-Acid 82%, Glycolic Acid 18%	Lorenz Surgical, Biomet Company, Indiana, USA	Edwards et al. [27]
Calciresorb	β-tri-calcium phosphate >96% Hydroxylapatit <4%	Ceraver Osteal, Roissy, France	Heini and Berlemann [28]
Calcibon	α-tri-calcium phosphate, calcium-hydrogen phosphate, calcium carbonate, precipitated hydroxyapatite,di-sodium hydrogen phosphate	Biomet Merck	Ooms et al. [29]; Hillmeier et al. [30]
Chronos	β-tri-calcium phosphate (100%)	Synthes, Oberdorf, Switzerland	Knop et al. [31]
Cerasorb	β-tri-calcium phosphate (100%)	Curasan AG, Kleinostheim, Germany	Zijderveld et al. [32]; Horch et al. [33]
Cerasorb M	β-tri-calcium phosphate (100%)	Curasan AG, Kleinostheim, Germany	Knabe et al. [34]
Tutogen bovin	sterile bovine bone	Tutogen Medical, Neunkirchen, Germany	Meyer et al. [15]
Tutobone human	sterile human bone	Tutogen Medical, Neunkirchen, Germany	Shin and Sohn [16]
Biobon	Tri-calcium phosphate, di-calcium phosphate dihydrate	Biomet Merck, Berlin, Germany	Linhart et al. [20]
Perossal	Nano crystalline hydroxylapatite 51.5%Calziumsulfate 48.5%	aap Implantate AG, Berlin, Germany	von Stechow, Rauschmann [35]

### Assay of Osteoclast Formation

To evaluate general effects of the bone substitute biomaterials on the differentiation of human osteoclasts, the development of cellular protrusions, as well as the fusion of mononuclear precursors, were examined morphologically every other day using a light microscope. Representative photomicrographs were taken at the beginning and after 14 days of differentiation. The pH value of cell culture media was determined twice a week using a MP 220 pH meter (Mettler Toledo, Schwerzenbach, Switzerland). After 28 days of incubation dentin and biomaterial chips were removed from the culture plates and subjected to the resorbability assay (see below). The cells were fixed with 3.7% formaldehyde (Merck, Darmstadt, Germany) for 5 min and air-dried for 2 min. In order to determine the number of osteoclasts and the number of nuclei per osteoclast at the end of incubation, fixed cells were stained with TRAP staining solution containing 5 mg Naphthol AS-MX Phosphate (Sigma), 500 µL *N-N*-Dimethylformamide and 30 mg Fast Red Violett LB Salt (Sigma) in 50 mL TRAP buffer (40 mM sodium-acetate and 10 mM sodium-tartrate in PBS) for 10 min. Cells with a positive staining for TRAP containing 3 or more nuclei were counted as osteoclasts. For quantification, the means of five representative fields of view in the pre-defined region of interest (Fig. 1, magnification 200×) per sample were determined by two independent investigators.

### Resorbability Assay

The biomaterial and dentin chips were washed with PBS to remove remaining cells. To visualize resorption lacunae, dentin chips were treated with 1% toluidin blue solution for the staining of collagen fibrils that are physiologically uncovered by the process of resorption (pit assay). The results were compared with a pit assay on day 0 of differentiation as an internal control, proving the complete absence of resorption lacunae on day 0. Unlike dentin, biomaterials do not necessarily contain collagen fibrils [12], so pit assays are not suitable for the determination of resorption lacunae on these materials. Since we could previously demonstrate a high comparability of toluidine blue staining and SEM (r = 0.996; p = 0.004) [36], biomaterials were analyzed using a Leo435vp scanning electron microscope (Leo, Oberkochen, Germany) at 150 mA and 20 kV after sputtering with gold in a sputter coater 180 auto device (Cressington, Watford, UK). The resorbed areas on dentin and biomaterials were quantified using the UTHSC-SA Image Tool (UTHSCSA San Antonio, USA). Resorption lacunae on biomaterials were identified by comparing the scanning electron microscopy (SEM) results of day 0 with that of day 28. Whole dentin chips and biomaterial samples were initially examined by light microscopy and SEM, respectively, to estimate the overall resorption area. Representative viewing fields were then defined by two independent investigators and photomicrographs were taken for subsequent quantifications. Finally, the resorbed area of either dentin or biomaterial was normalized to the number of TRAP+ multinucleated osteoclasts present in the respective wells.

One aim of the study was to determine the clinically relevant question of which of the tested materials might be preferentially resorbed by osteoclasts in comparison to the surrounding bone tissue. Therefore, the relative resorption coefficient (RRC) was calculated for each material, which represents the ratio between resorption on biomaterial and resorption on dentin [9]. A RRC of 1 indicates a balanced resorption of dentin and biomaterials, whereas a value smaller than 1 indicates lower osteoclastic resorption of the biomaterial surface compared to resorption of the dentin surface.

### Statistics

All cell culture experiments were done with groups of at least n = 5 and repeated at least three times with cells from two different donors. Statistical analysis was done using ANOVA with Bonferroni post-hoc test and Spearman correlation using IBM SPSS Statistics Version 20.0 (SPSS Inc., Chicago, IL, USA). The probability of a type I error was set to 5% (alpha = 0.05). Error bars represent standard deviation (SD).
